# Enhancing the Bystander and Abscopal Effects to Improve Radiotherapy Outcomes

**DOI:** 10.3389/fonc.2019.01381

**Published:** 2020-01-08

**Authors:** Virgínea de Araújo Farias, Isabel Tovar, Rosario del Moral, Francisco O'Valle, José Expósito, Francisco Javier Oliver, José Mariano Ruiz de Almodóvar

**Affiliations:** ^1^Centro de Investigación Biomédica, Instituto Universitario de Investigación en Biopatología y Medicina Regenerativa, PTS Granada, Granada, Spain; ^2^CIBERONC (Instituto de Salud Carlos III), Granada, Spain; ^3^Instituto de Parasitología y Biomedicina “López Neyra”, Consejo Superior de Investigaciones Científicas, PTS Granada, Granada, Spain; ^4^Complejo Hospitalario de Granada, Servicio Andaluz de Salud, PTS Granada, Granada, Spain; ^5^Departamento de Anatomía Patológica, Facultad de Medicina, Universidad de Granada, PTS Granada, Granada, Spain

**Keywords:** experimental radiotherapy, cell loss, mesenchymal cells, bystander effect, abscopal effect, exosomes, mesenchymal cell enhancement ratio

## Abstract

In this paper, we summarize published articles and experiences related to the attempt to improve radiotherapy outcomes and, thus, to personalize the radiation treatment according to the individual characteristics of each patient. The evolution of ideas and the study of successively published data have led us to envisage new biophysical models for the interpretation of tumor and healthy normal tissue response to radiation. In the development of the model, we have shown that when mesenchymal stem cells (MSCs) and radiotherapy are administered simultaneously in experimental radiotherapy on xenotumors implanted in a murine model, the results of the treatment show the existence of a synergic mechanism that is able to enhance the local and systemic actions of the radiation both on the treated tumor and on its possible metastasis. We are convinced that, due to the physical hallmarks that characterize the neoplastic tissues, the physical–chemical tropism of MSCs, and the widespread functions of macromolecules, proteins, and exosomes released from activated MSCs, the combination of radiotherapy plus MSCs used intratumorally has the effect of counteracting the pro-tumorigenic and pro-metastatic signals that contribute to the growth, spread, and resistance of the tumor cells. Therefore, we have concluded that MSCs are appropriate for therapeutic use in a clinical trial for rectal cancer combined with radiotherapy, which we are going to start in the near future.

## Introduction

In clinical oncology, each patient is different. Therefore, the treatment should also be different; that is, each patient needs a specific treatment adjusted to their characteristics and the prognosis of the illness.

For most neoplastic diseases, the prognosis of the disease is a function of a small number of variables. Although the choice of these variables is supported by a broad medical consensus and it is assumed that each treatment is considered to be the most appropriate to achieve a cure, the number of therapeutic failures that result constitutes a medical problem of singular importance.

Currently the treatment of cancer patients is decided on the basis of the size of the tumor, the status of the loco-regional lymphatics, the presence or absence of distal disease, the histological type, and the general health state of the patient. Once the necessary values are known, the patients are classified (the staging) into well-defined clinical categories (1). This classification is so that the physician has a general approach to the prognosis of the illness suffered by the patient being treated, and that the treatment proposed is most appropriate and above all offers the patient the necessary information to decide and consent to how he/she wants to be treated.

Ionizing radiation is widely and effectively applied in oncology. However, due to dose limits, a complete tumor cure cannot be achieved for many tumors and localizations. Despite the advanced radiotherapy facilities and therapeutic methods that are currently available, high doses of radiation might still induce, fortunately only on rare occasions, early and late side effects of severe magnitude. Unacceptable normal tissue reactions persist as the limiting factor for administering a tumoricidal dose in radiotherapy. Moreover, the frequent presence of clinical and/or hide-metastatic foci in distal organs is beyond the range of the treatment and is a death threat for the patients. Therefore, research searching for progress in the control of metastatic disease is a target of major interest.

The previous paragraph reveals that both the study of the extension of the neoplasms and the prediction of the probabilities of tumor control or complications after therapy are based on techniques that are imperfect, imprecise, and insufficient. Indeed, when the results of therapy in groups of patients classified to be at the same stage are studied in the long term, a variability of response is found, which is impossible to predict (1–6).

The evolution of ideas and the study of successively published data have led us to imagine a new biophysical model for the interpretation of tumor response to radiation. In its development, we have shown that when human-umbilical cord mesenchymal stem cells (MSCs) and radiotherapy are administered simultaneously in experimental radiotherapy on xenotumors implanted in a murine model, the results of the treatment show the existence of a synergic mechanism that is able to enhance the local and systemic actions of the radiation both on the treated tumor and on its possible metastasis. We are convinced that due to the physical hallmarks that characterize the neoplastic tissues, the physical–chemical tropism of MSCs, and the widespread functions of macromolecules, proteins, and exosomes released from activated MSCs, the combination of radiotherapy plus MSCs used intratumorally has the effect of counteracting the protumorigenic and pro-metastatic signals that contribute to the growth, dissemination, and resistance of the tumor cells.

Therefore, we have concluded that the administration of MSC enhances the therapeutic effect of radiotherapy *in vivo* and does not produce toxic effects, indicating that they could be used as an adjuvant treatment for cancer, increasing the therapeutic effect of radiotherapy on the tumor as well as on possible tumor-metastatic foci.

The three objectives of this study are:

- to propose a biophysics model that includes the classic radiobiological concepts together with the bystander and abscopal effects in a single picture.
- to summarize results of our *in vivo* studies that demonstrate of the synergist effect of radiotherapy combined with mesenchymal cell therapy in the treatment of xenotumors.
- to suggest that exosomes and proteins secreted by the activated-mesenchymal cells are responsible for the enhancement of radiotherapy action on the tumor, including the abscopal effect on tumor-metastatic foci.

## The Role of Radiobiology

The cellular consequences of direct radiation-induced DNA damage, producing lethal and potentially lethal damage to DNA, can be described by radiation cell survival models (7). Nevertheless, although we agree with Brown et al. (8), who suggested that, for the most part, the universally accepted radiobiology ideas of the 5 Rs (9) are enough to describe the clinical data and the isoeffect or tolerance calculations, we are convinced that the results obtained from the application of the LQ model (10, 11) in clinical studies through the calculation of biologically effective doses (BEDs) are absolutely correct, and that this model has also been successfully used, even with stereotactic radio-surgery (SRS), intraoperative radiotherapy (IORT), and stereotactic body radiotherapy (SBRT), although with the probable exception that, for some tumors in which high doses of irradiation may produce greater anti-tumor immunity (8), the role of the 5 Rs of radiotherapy is clearly different in these cases (12).

However, considering that the LQ model can explain neither the bystander effects (13–15), nor the variation of damage processing and tissue remodeling in the pathogenesis and severity of the of the late effects of radiation (16–18), nor the abscopal effects that can be intermediated principally by immune cells such as the T cells (19), it is clear that the models so far used to interpret the relationship between cell radiosensitivity and clinical radio-response are unable to explain all the effects of radiation in some circumstances and a more general radiobiological model appears to be mandatory (6, 20).

## We Must Understand the Whole Response of Tumor and Normal Tissue to Radiotherapy

The happening of hyper-radiosensitivity at low radiotherapy doses (13) and the bystander effect (14–16) exemplifies that reactive molecular signaling and repair activity regulate the equilibrium of irradiated potential lethally damaged cells between radiation cell killing and cell survival, and this communication between irradiated and out-of-target cells can affect tumor cells, reducing their surviving fraction (17, 18).

Mounting data suggest that radiotherapy also recruits biological effectors away from the treatment field and has systemic effects (19, 20).

Consequently, in our view, non-target radiotherapy action could be thought as the complete immunological reaction of the tumor (21–25) and health tissues (6, 26) to the stress caused in the irradiated volume (27) that results in enhanced levels of DNA lesions (21), chromosomal aberrations (28), alterations in transcript levels and gene expression (29–32), and finally cell death (18, 33). The major question, however, is how to combine diverse information (clinical, imaging, and molecular data) in an algorithm to offer specific clinical information that precisely and significantly estimate patient outcomes as a function of potential therapeutic decisions (34).

We consider that neoplastic stem cell survival after radiation treatment be determined by (a) the effects of radiation-induced cellular damage (linear-quadratic model) and (b) the out-of-target bystander and abscopal interaction produced by free radicals, antigen–antibody interaction (19), and death receptor–ligand interaction (18, 35, 36).

### The Biophysical Model

Assuming that the targeted action of radiation on the cell DNA and the non-targeted (bystander or abscopal) actions on cell survival are independent as has been proposed recently (37), our previously published model (35, 38) defines the final surviving fraction as the product of the surviving fraction produced by the targeted interaction of radiation with the tumor cells and the cell surviving fraction on tumors and metastatic foci through the short-range and long-range bystander effects that are promoted by the radiation treatment (18, 35).

**Figure 1:**
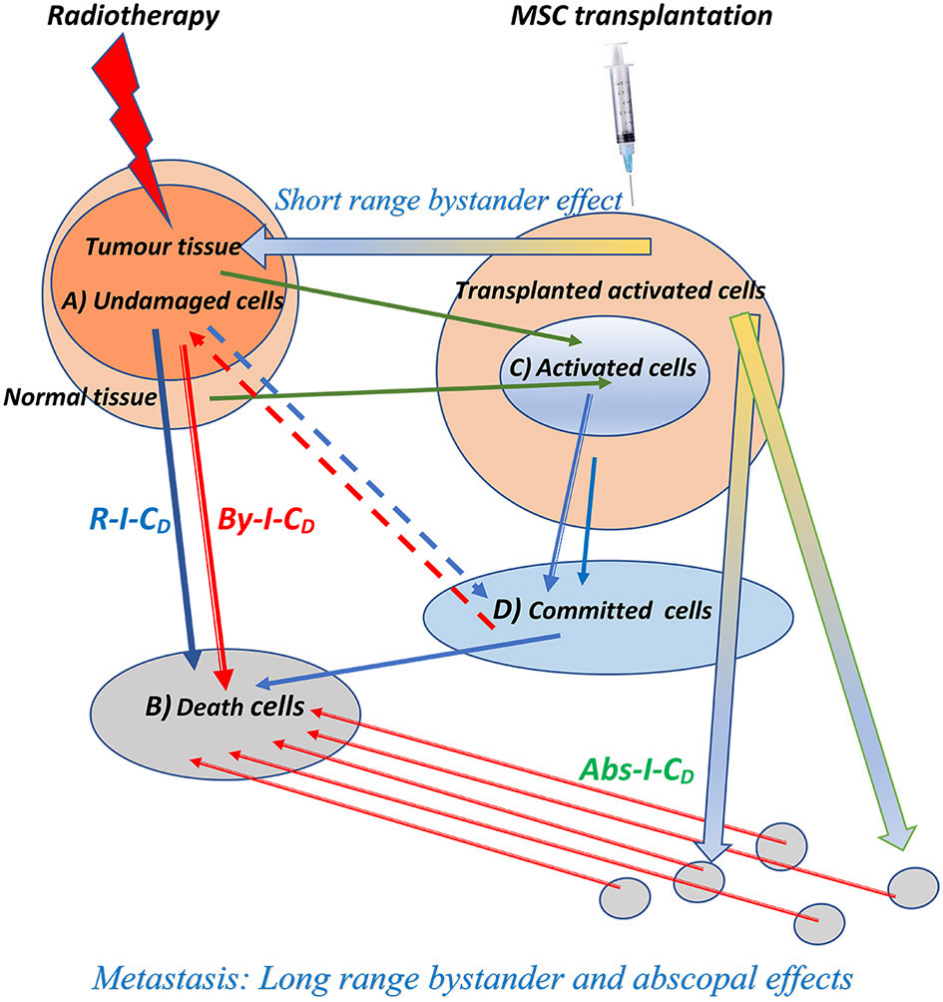
Schematic representation of the biophysical model for direct, bystander, and abscopal actions of the radiotherapy and the enhancement produced by the addition of cellular therapy through mesenchymal cell transplantation simultaneously applied with radiation therapy. ***Rt-I-CD***, radiation-induced cell death on tumor cells; ***By-I-C***_***D***_, short and long-range bystander-induced cell death on tumor cells; ***Abs-I-C***_***D***_, abscopal-induced cell death on metastatic foci. The three types of induced cell death are enhanced by the combination of radiotherapy and cellular therapy, and the compartment of *activated cells* responsible for bystander and abscopal effects, labeled with (C) in the figure, may be enlarged by mesenchymal cell transplantation.

Based on these concepts, we have described that, after radiation, cells in the therapeutic volume can be classified into four compartments (Figure 1) that we briefly update here:

#### Undamaged Cells (A)

Survival response of cells after each fraction of dose, which should be controlled with consecutive irradiation treatments.

#### Dead Cells (B)

This is the lethal-lesion compartment in Curtis's model (39) that arise from the targeted and non-targeted action of radiation on DNA, and from the bystander and abscopal immunological cell death promoted by the action of activated cells (38, 40) and death cells (35, 40–42) on other tumoral cells belonging to tumor process.

#### Activated Cells (C)

Cells that are either slightly damaged or have been able to restore their lesions to a level of residual damage compatible with survival. These cells might turn out to be an effective source of cytokines (38), macromolecules (43), exosomes (44, 45), reactive oxygen species (46), and reactive nitrogen species (18), and/or could suffer phenotypic changes to express hide-antigens in the tumors, which allow the triggering of the pro-immunogenic effects of radiotherapy on the tumors (19, 20, 47), with none of these possibilities being exclusive of the others, indeed all of them might affect the local and distal burden of tumor cells (48) and be the cause of the bystander and abscopal components of the radiation immunologically induced cell death on local and distal foci of the tumors (35).

As we will explain below, this compartment may be enlarged by human-umbilical cord MSC transplantation (38, 44, 49).

#### Committed Cells (D)

This compartment corresponds with the potentially lethal lesions in the LQ Equation (7, 39); cells in this compartment can flow back to compartments ***(A)*** or ***(B)*** through proper repair or binary misrepair.

### Operational Terms

As operational purpose, we considered:

#### Short-Range Bystander Effect

It is generally accepted that the use of ionizing radiation to a treatment volume that contains the tumor causes effects that go beyond radiation-induced cell death (14), revealing intracellular transmission that implies the gap-junction intercellular connection and ends in cell death, enhanced amounts of DNA double-strand breaks, induction of chromosomal aberrations, and/or alterations in transcript RNA levels and gene expression (50).

#### Long-Range Bystander Effects

Results obtained from “*in vivo*” irradiated tumors suggest that tumors may exert their influence far beyond its own microenvironment to spread peritumoral region and tissues far away from a tumor. The long-range bystander effect is generated by cytokines, macromolecules, and exosomes liberated into the extracellular space (38, 40, 44) that, through the lymphatic or vascular systems, might substantially alter conventional expectation in radiotherapy by yielding loco-regional positive effects (35).

#### Abscopal Effect

The abscopal effect is an anti-tumor radiation consequence seen in metastatic disease placed far away from the irradiated tissue. High-dose ablative radiotherapy results in release of debris of tumor cells containing molecules that may be immunogenic (51). Therefore, radiotherapy could imitate the effect of vaccination, as an unconventional method to present tumor antigens making cancer cells more receptive to T cell-mediated cytotoxicity (52). This effect could be associated first with the larger than usual effect of single doses by standard models (53), thus facilitating excellent local control rates, second to the unexpected abscopal effect. In this sense, new original associations of RT with immunotherapies have been designed to reverse tumor immune-related radioresistance (54), and the reactivation of the anti-tumor immune response can be considered as the 6th R of clinical radiobiology (36), opening an exciting field in patient treatment.

It is important to underline that unlike an increased reply concomitant with an escalation in radiation dose, the bystander reaction reaches a saturation level at comparatively low doses (18, 50).

### The Mathematical Model

A key feature of bystander responses, as opposed to direct irradiation effects, is the dose–response relationship. Instead of a continuously increased response related with an increase in dose, the bystander response turns out to be saturated at low doses. This might indicate a receptor–ligand interaction, which we took as our original hypothesis (18), with the characteristic of being simultaneously dynamic and reversible. The same kinetic mechanism could be used to describe the antigen–antibody interaction.

Assuming the radiation and bystander consequences on tumor cell survival to be independent (38, 44), the whole response of the tumors and their metastatic foci to radiation therapy might be expressed as the product of the probability of radiation tumor cell death times the probability of cell death through bystander or abscopal effects (18, 37).

Data now indicate that, as well as these targeted DNA damage dependent effects, tumor cells and normal tissue-irradiated cells (activated cells) and immunological cell death also transmit signals to their adjacent cells (35). Here, we think that clonogenic cell survival ***S*** after radiation therapy depends together with the direct effects on DNA through radiation interaction ***S***_***RT***_ and bystander and abscopal communication **Π** ***S***_***By***_. Thinking that DNA damage caused by radiation and short- and long-ranged bystander effects on tumor cell survival are independent, the whole tumor response may be said as:

(1) $$\begin{array}{l} S=S_{RT}\cdot \overset{n}{\underset{i\;=\;1}{\prod }}S_{By\left(i\right)} \end{array}$$

where the first term of the product of cell survival calculated using linear-quadratic model represents the pure RT action on the irradiated tumor and the second term, which begins with the Π symbol, is the product from *n* = 1 to *n* = *n* of the cell death probabilities resulting from each one (*i* = 1…*i* = *n*) of the out-of-target cell death actions (bystander and abscopal effects) promoted for the combined treatment (RT + MSCs) that was used in our last papers. A set of these possibilities has been summarized here in the point section Activated Cells (C).

Following the same reasoning that we indicated in our previous paper (35), this entails that the chance of cell survival depends on both the direct radiation effect (the LQ model) and bystander effects, with these effects also being a phenomenon composed of long- and short-range bystander actions, whose importance, at least in experimental RT, may be estimated.

The final values of tumor cell survival (*S*) suggest that the lethal effects of radiation on tumor cells can be significantly enhanced by unanticipated interactions between live cells with the secretome of activated cells (14, 44) or with the molecules released after immunological cell death (42).

This model helps us to comprehend how anticancer treatment may have an additional and significant effect in that the radiation-activated MSC^*^ cell response could be important for therapy to be successful due to the fact that the survival of tumor cells interconnecting with irradiated and activated cells is reduced.

## The Long-range Bystander Effects and the Abscopal Effect of Radiotherapy

Anti-tumor consequences beyond the radiation field have been identified (43, 47, 55–61) and the regression of remote metastasis after tumor radiotherapy has been recently described in human melanoma skin cancer (48, 62, 63) and other tumor locations (64, 65).

Over recent years, the abscopal mechanism has been clarified by the effort of several investigators, including Formenti and Demaria (19, 20), who revealed that this activity was probably facilitated by the immune system leading to immunogenic cell death, a mechanism that implicates dendritic cells, T regulatory cells, and suppressor cells as key intermediaries. Radiation therapy sensitizes unresponsive tumors to the anti-neoplastic action of antibodies that target the inhibitory receptor CTLA-4 on T cells (66). Multiple studies have demonstrated that radiotherapy can increase the efficacy of anti-PD1 therapy by priming and recruiting more anti-tumor effector T cells (67, 68) and recently it has been suggested that the addition of immune checkpoint inhibition with local radiotherapy might increase local and distant metastatic control and, in the end, the clinical results of disease control in patients with oligometastatic cancer (69). Moreover, the idea to generate an integrated clinical and molecular categorization of metastases along the spectrum of disease is very interesting, because this approach may perhaps influence the staging and treatment of patients with cancer diseases (70).

Golden and colleagues (42) were the first to prove that abscopal responses can be consistently identified in patients with confirmed solid metastatic cancer treated with radio- and immune-therapy. The combined treatment with and the granulocyte-macrophage colony-stimulating factor generated clear abscopal responses in certain patients with metastatic diseases, and this finding signifies a hopeful advance to establishing an *in situ* anti-tumor vaccine (71). Recently published results prove that radiotherapy in combination with the CTLA-4 blockade (72) or the PD1 blockade (68) produces systemic effects in patients with cancer. The early response in the TCR clonal dynamic detected in responders is coherent with a change and increase of the tumor-directed TCR repertoire provoked by radiation therapy and its study in representative cases means that increase of a huge amount of tumor-specific T cell clones in peripheral blood and their presence over time correlated well with the occurrence of abscopal outcomes (72). In spite of the growing number of clinical studies examining the ability of radiation to improve immunotherapy, clinical proof that it transforms cold tumors with little to no immune response into responsive ones remains elusive (66).

Nevertheless, it seems clear that reasonable combinations of immunotherapy with RT may dramatically change the model of care for many tumor types in the following decade (73).

## Mesenchymal Cells as Biological Response Modifiers

It is generally recognized that MSCs can be found commonly in numerous tissues and are not limited to those of mesodermal origin, such as bone marrow, adipose, muscle, and bone (74). On the other hand, it has recently been revealed that *in vitro* differentiation of human MSCs is linked by an augmented sensitivity to apoptosis, which is in significant divergence to undifferentiated MSCs, which are moderately resistant to irradiation or temozolomide-induced DNA damage (75). We have demonstrated that MSCs are relatively sensitive to low-LET irradiation and very resistant to the bystander effect produced by the culture medium of irradiated cells (18).

Stem cell knowledge has also become the basic element in regenerative medicine (76, 77).

It is an exciting idea that inhibiting the mechanism that facilitates the bystander effect can give rise to therapeutic approaches that stimulate the radio-sensitivity of cells or protect healthy tissue against the damaging effects of ionizing radiation (78). Previous reports suggested a protective role for MSCs when combined with RT (79, 80). In effect, study on mesenchymal stem cell therapy for wounded and unhealthy tissues, involving the intestines, has been highly encouraging. Therapy with bone-marrow-derived or vascular-wall-derived MSCs protects the lung tissue from radiation-induced vascular damage and antagonizes the metastatic potential of circulating tumor cells to formerly irradiated lungs (81).

The use of human grade MSC is challenging and must fulfill EMA or FDA requirements to regulate autologous adult stem cells for therapeutic application. This has been widely summarized and discussed (82, 83) and we know that MSCs, commonly mentioned to as MSCs or mesenchymal stromal cells, are a varied population of cells that must be properly characterized. To clarify this controversial aspect, different papers have been published in the latest years (84–86) and contribute to the understanding of the composition of MSC-based products and provide the way to assess their *in vitro* and *in vivo* bioactivity.

Due to their properties, MSCs might be suitable as a therapeutic tool for handling radiation-induced normal tissue injury (84, 87). Numerous papers have demonstrated that administered either intraperitoneally or intravenously, MSCs effectively home onto primary tumors and their metastases (85, 86). Moreover, before supporting tissue repair functions, MSCs first organize the microenvironment by controlling inflammatory processes and releasing a variety of growth factors in reaction to the inflammation process (88). Due to their trophic, paracrine, and immunomodulatory functions, they may have the highest beneficial impact *in vivo* (89). However, the amount of MSCs that engraft into damaged tissues might not be enough to explain their robust protective effect.

The therapeutic efficacy of transplanted MSCs seems to be unconnected to the physical proximity of the transplanted cells to damaged tissue. Thus, we believe that the predominant mechanism by which MSCs contribute in tissue repair might be related to their paracrine activity, and in this way, it is also possible to think of the additional use of MSCs as an adjuvant to support and complement other therapeutic options as has recently been recently proposed (65, 90).

## Is it Possible to Widen the Bystander and Abscopal Radiotherapy Effects?

MSCs have been studied for the treatment of cancers as they are able to home onto tumors and come to be incorporated into their stroma. Moreover, MSC homing is enhanced after radiotherapy (45). MSCs can both suppress or stimulate tumor progression (91–93). It has been described that the bioactivation of MSCs may be achieved by different treatments and the molecules secreted by the activated MSCs (MSCs^*^) could have an influence on a variety of immune cell lineages and establish a beneficial field (40).

We have recently shown that optimal bystander and abscopal effects can be obtained using MSCs plus RT administered on an experimental murine model with two xenotumors symmetrically placed in the upper region of both the rear legs, with only one of them being treated with radiotherapy (38, 44).

In a recently published article, the influence of MSC cell therapy on the progress of solid tumors using an orthotopic cancer model of human colorectal cancer cells has been studied, as well as in an immunocompetent rat model of colorectal carcinogenesis representative of human pathology (49). In their results, the authors show that MSC administration to immunocompetent rats treated topically with methylnitronitrosoguanidine (MNNG), a strong carcinogen, reduced the growth of the tumors and improved overall survival. In this experimental cancer model, the MSCs have strong action on colon cancer growth by altering the immune component of the tumor microenvironment. In an important concordance with our research (38, 44), when MSCs were administered after therapy of colorectal cancer (CRC) with fractionated irradiation, MSCs reduced tumor growth, extended animal survival, and reduced the presence of metastatic foci.

The MSCs also protected healthy tissue from radiation damage by rising the levels of growth factors, reducing fibrosis, and facilitating intestinal recovery (49).

Taking into account both the previous reports and our own experience and research on the extraordinary abilities of proliferation (94, 95), secretion (44, 96), and differentiation (95) of the umbilical cord mesenchymal cells that we have investigated (38, 44) and used in combination with radiotherapy in recent years, we have developed the following hypothesis:

“Radiotherapy may not only be a successful local and regional treatment but also a novel systemic cancer therapy” (38).

To check this hypothesis, we used a set of human cancer cell lines implanted in NSG mice as xenotumors and MSCs obtained from human umbilical cord stroma. We have investigated the tumor response to direct irradiation (2Gy low-LET radiation fraction administered once a week for 5–6 weeks) and, in the non-irradiated contralateral tumor, the tumor sensitivity to the bystander effect.

In our experiments, mice with tumors larger than 60 mm^3^ were treated with an intraperitoneal administration of 10^6^ MSC once a week for 5–6 successive weeks (38, 44). The day after each cellular therapy, one of the four groups of mice was randomly chosen to have one of their tumors irradiated. Ionizing radiation was delivered by X-ray TUBE (YXLON, model Y, Tu 320-D03) as explained previously (38, 44). The treatment was repeated once a week for a total of 5–6 weeks. The other mice groups were treated with exclusively RT or exclusively MSC. The mice in the control group received no treatment (38, 44).

We have proved that tumor cell loss induced after treatment with radiotherapy enhances with the therapeutic combination of RT and MSCs, when compared to RT alone, in the three cell lines (A375, G361, and MCF7) used, and also that, through the bystander and abscopal effect, the therapeutic combination (RT + MSC) had a positive effect on the tumor-volume reduction of the contralateral, untreated tumor (Table 1). When the cell line used had metastatic potential, the combination (RT + MSC) produced a reduction in the microscopic number of metastasis in the internal organs of mice with A375 xenotumors (44). These results prove conclusively that the combination of MSC + RT produces a synergic, bystander, and abscopal effect.

**Table 1:** Characteristic growth kinetics parameters of the treatment of xenografts implanted in NSG mice on control and MSC + RT groups.

**Parameter**	**Tumor cell line**					
	**G361**		**A375**		**MCF7**	
	**RT**	**MSC + RT**	**RT**	**MSC + RT**	**Control**	**MSC + RT**
T_D_ (days)	11.5 (CI: 10.6–12.6)	22.5 (CI: 18.7–28.1)	7.6 (CI: 5.3–5.7)	8.5 (CI: 8.1–8.9)	17.6 (CI: 17.2–18.2)	38.9 (CI: 32.3–47.5)
CL (% days^−1^)	47.0	72.3	9.6	18.8	–	55.9
MSC-ER	1.6		2.0		Not calculated	
T-t-G (days)	60.8	91.1	32.8	36.6	195.0	422.3
Mx	No		Yes: 1.0 ± 0.4	Yes: 0.4 ± 0.1	No	
% reduction Mx index: 60%; *P* = 0.002						

*T_D_, tumor volume doubling time (days); CL, cell loss factor in a treated tumor compared to a control tumor (%**·**days^−1^); MSC-ER, radiotherapy mesenchymal cell enhancement ratio = (cell loss produced by the combination RT + MSC)/(cell loss produced by RT alone); T-t-G, time-to-tumor growth (days) to reach a volume of 2.0 ml; Mx, Metastasis index: % of decrease in the histological identification of microscopic metastasis in the MSC + RT group, compared to the control group; CI, confidence interval*.

In Table 1, notice the differences in the tumor volume doubling time values (T_D_) corresponding to different cell lines treated with RT (from 7.60 to 17.60 days) and observe, also, the differences between the control and MSC+RT groups for each of the tumor cell lines implanted as xenotumors (from 22.5 to 38.9 days) and the gains derived from the addition of MSC to the RT treatment, measured as the mesenchymal cell enhancement ratio (MSC-ER), ranged between 1.60 and 2.00 and more than 3.00 for A375 in our last paper (44) designed to evaluate the anti-metastatic potential of MSCs combined with RT, when the tumor volume was followed only in the first 14 days.

It is important to analyze that the time-to-tumor growth to a volume of 2 ml reached an increase in time ranging between 12% for A375, the most aggressive cell line, and 117% for MCF7, the least aggressive. For details on the mathematical model used [see (38, 44)]. It is important to highlight that G361 and A375 are human melanoma cell lines, whereas MCF7 is a cell derived from a human breast cancer.

We define cell loss factor as CL = 100·[1 – T_D_(*control*)/T_D_(treatment)]; in which T_D_(treatment) is the volume doubling time in each of the treatment groups: MSC +RT and RT.

MSC-ER: the mesenchymal enhancement ratio is the ratio between the cell loss corresponding to the combined treatment divided by the cell loss corresponding to the treatment with radiotherapy alone.

CL: in the cell-loss factor, the following are included: (i) all the types of cell death, (ii) lengthening of the mean cell cycle duration produced by the treatment, and (iii) cells that have a null or limited growth potential due to misrepair of damage or because they have been involved in a differentiation process.

The abscopal effect has been estimated by the reduction of metastasis index that was 60% in the A375 cell line, with the difference between the control and RT + MSC groups being statistically significant (*P* = 0.002). In our experiments, A375 is the only cell line that has showed metastatic potential. It is very important to note that the amount of metastatic foci observed in the internal organs of the mice treated with MSC + RT was 60% fewer than in the mice treated with RT alone (44).

Moreover, in our last paper (44) (supplementary materials), we demonstrated that MSC, previously activated with 2 Gy low-LET radiation dose (MSC^*^) and used after tumor radiotherapy as adjuvant cellular therapy, retained a wide cytotoxic activity that affected the volume of the xenotumors treated, thus enhancing the therapeutic effect of radiotherapy in a similar level to that we have communicated previously (38, 44). Using these MSC^*^-activated cells, we found that when the tumors implanted in mice were first treated with radiotherapy and then treated immediately after the end of RT with infused intraperitonially MSC^*^ activated, the tumors treated in this way significantly reduced their tumor growth rate compared with both control mice and mice treated with radiotherapy alone.

Accordingly, the results obtained in our study regarding the tumor doubling time (T_D_) values were different among the groups, being longer for mice treated with RT + MSC^*^ (8.46 days), compared with the control and RT groups (6.87 and 7.60 days, respectively).

Mesenchymal enhancement ratio (MSC-ER) is the ratio of tumor effect produced by the combination of radiotherapy plus MSCs therapy (RT + MSC^*^) divided by the tumor effect produced by exclusive radiotherapy. By means of doubling the time values, we have calculated the MSC^*^-ER values as the proportion of cell loss *C*_*L*_ (38) produced by RT + MSC^*^ treatment, compared to the cell loss *C*_*L*_ produced by radiotherapy treatment alone and demonstrated that activated MSC^*^ potentiated the radiotherapy effect when infused into tumor-bearing mice with a MSC^*^-ER of:

(2) $$\begin{array}{l} {\text{MSC}}^{*}-\text{ ER}=\frac{{\text{C}}_{\text{L}}\;({\text{RT }+\text{ MSC}}^{*}\text{ treatment})}{{\text{C}}_{\text{L}}\;(\text{RT treatment})}=\frac{18.\;8\%}{9.6\%}=1.95 \end{array}$$

This result fits with previous results summarized in Table 1 and proves that the combination (RT + MSC^*^) improves the therapeutic efficiency respect to RT alone (both in tumor and metastatic control) through enhancing short- and long-range bystander and abscopal effects. For more details on the mathematical model applied [see supplementary materials in (37)].

## Cellular Therapy With MSCs: a Problem for Anti-Tumor Therapy?

MSCs exist in many tissues and are recognized to actively be recruited to primary tumors and metastasis and also to other locations of normal tissue damaged, where they take part in wound repair. Tumors can be thought of as “wounds that never heal” and, in reply to signals from the neoplastic tissue, the MSCs can exhibit a marked tropism that might contribute to tumor growth promotion by several mechanisms that have been reviewed recently (97).

Tumors continuously recruit cells from the tumor microenvironment and become important elements of the tumor volume, interchanging proper signals that might acquire aggressive phenotypes of carcinoma cells and establish a complicated situation that concludes in metastasis (98). Recently, it has become apparent that tumor-associated MSCs have an effective role in tumor induction, promotion, growth, and metastasis (99), and although the tumor microenvironment is constituted of numerous cell types including tumor, stromal, endothelial, and immune cell populations, it appears clear that, under the influence of these cells, MSCs acquire different functional phenotypes that promote tumorigenesis (100), permitting the tumor to avoid immune clearance or impeding effectiveness through the acquisition of a chemotherapy and radiotherapy resistance mechanisms (101). On the other hand, it has been described that, in an inflammatory situation, resident tumor MSCs strikingly enhanced tumor growth by engaging monocytes/macrophages in comparison to bone marrow MSCs (102, 103) and exosomes present in the cancer cell secretome might be the principal agent able to modify the normal MSC cell phenotype toward a malignant one (104).

Nevertheless, it is still controversial whether this innate tropism of MSCs toward the tumors and metastatic foci is linked with cancer promotion or suppression (105), and it has been suggested that a better understanding of the interactions between cancerous cells and stromal components of tumor microenvironment is important to allow progress in the development of more specific and useful therapies in cancer (99, 100, 106).

## Exosomes Secreted From MSCs Have a Totally Different Effect From the Exosomes Released From Tumor Cells

Cancer cell-derived exosomes have been shown to participate in the key steps of the metastatic widening of a primary tumor, ranging from oncogenic reprogramming of malignant cells to the formation of pre-metastatic niches (107) and this mechanism may be facilitated by RT under certain conditions (108, 109) or facilitated by released mir-939 in exosomes that, once internalized in endothelial cells, play a protumorigenic role for metastatic spread in association with triple-negative breast cancers (110).

By studying the exosomes and microvesicles released by tumor cells into the extracellular medium, we have been able understand that exosomes from tumor cells spread through the biological fluids and support tumor growth and metastasis formation (111, 112). There are several examples that confirm this hypothesis; for example, it is well-known that the process of cancer cell migration into the normal tissues and invasion-promoting effects may be due to cancer-cell-derived exosomes (113, 114). After release, the exosomes are taken up by neighboring or remote cells facilitating tumor progression and the miRNAs confined within the exosomes modify such processes as interfering with tumor immunity and the microenvironment, suggesting that exosomal miRNAs have a noteworthy role in regulating cancer progression (115).

Pancreatic ductal adenocarcinoma (PDAC) is an exceptionally aggressive tumor, characterized by a high metastatic potential, even at the point of diagnosis; in a recent paper (116), using proteomic studies, it has been shown that it is possible to identify the impact of exosomes on the Kuppfer cells in the liver, which may function to organize this organ for metastatic occupation. Recently, the exosome-mediated transfer of pyruvate kinase M2 (PKM2) from PCa cells into bone marrow stromal cells (BMSCs) has been identified as a new process through which primary tumor-derived exosomes stimulate premetastatic niche development (117).

Other published results (118) suggest that exosome-mediated discharge of tumor-suppressor miRNA is selected in tumor evolution as a mechanism to organize the activation of a metastatic cascade (119). The load of exosomes is given for the parental cells and the circumstances in which they deliver them, which implies that circulating miRNAs in exosomes have the ability to serve as prognostic and predictive biomarkers (120).

However, exosomes derived from MSCs play a completely different role, and previous reports have suggested a protective role for MSCs when combined with RT. Indeed, therapy with bone-marrow-derived MSCs or vascular-wall-derived MSCs protects the lung tissue from radiation-induced vascular dysfunction and antagonizes increased metastases of circulating tumor cells to previously irradiated lungs (81).

Exosomes produced by MSCs have been demonstrated to contain antiapoptotic miRNAs to improve epithelial and endothelial wound healing and angiogenesis, and to include growth factor receptor mRNAs, well-known to facilitate wound recovery and safeguard the intestines from experimental necrotizing enterocolitis (121). Results of the research on mesenchymal stem cell therapy for wounded and unhealthy tissues, including the intestines, have been highly promising (79, 80) and MSCs may be considered as a therapeutic tool to deal with radiation-induced tissue damage (87).

It is important to underline that the group of Chapel et al. (122) has initiated a phase 2 clinical trial (ClinicalTrials.gov Identifier: NCT02814864) for the treatment of severe adverse effects for patients receiving radiotherapy for prostate cancer and that this clinical trail is supported by numerous papers focused on the use of MSCs for alleviating the side effects on normal tissues after radiation therapy (123–125).

However, the biodistribution and the mechanism involved in the control of colateral side effects are not well-known, although there are also some reports directed to investigating this problem in more depth. But we do know that in an undamaged mouse, exogenous intravenously injected MSCs quickly accumulate within the lungs and are cleared from this site to other tissues, such as the liver, within days (126). Nevertheless, the quantity of MSCs that are uptaked for the injured tissues may not be sufficient to explain their strong protective effects.

Moreover, in a cancer rat model used to study the treatment of chemical-induced colorectal cancer (CRC) previously cited (49), it has been demonstrated that exogenous MSCs, although only briefly found in the colon tissue of treated animals, were able to alter the immune profile of the tissue microenvironment as far as 1 year after the last MSC administration, possibly due to polarization of resident MSCs and immune cells.

To sum up, it is generally accepted that MSC-derived microvesicles and exosomes have been proposed as a novel mechanism of cell-to-cell communication that permits the transmission of functional proteins or genetic material via mRNAs and microRNAs upon cell activation that may encourage a new approach for repairing acutely damaged organs by virtue of the exclusive MSC tropism for the injured tissue, as well as their paracrine action in nature and facilitated through the decrease of inflammation and enhancement of tissue repair (127).

On the other hand, our *in vitro* and *in vivo* results show that TRAIL and DKK3 are molecules delivered by mesenchymal cells that, as consequence of the cell treatment with 2 Gy low-LET gamma radiation, are released to the extracellular space where they can work as signaling molecules to yield tumor cell death (38, 44). The ability of MSCs to release TRAIL to culture medium that inhibits the growth of human cancer cells has recently been confirmed (128). Exosomes and microvesicles also appear in the extracellular medium of cell cultures that are quantitatively, qualitatively, and functionally different if they are removed from the MSC medium or from the activated MSC medium (44).

Together, all these results indicate that the administration of MSCs might be a safe and innovative therapeutic alternative to heal normal tissue after cancer radiotherapy (49).

## Annexin A1 as a Candidate for Enhancing Radiotherapy

When we examined the exosome load before and after the activation of MSCs with RT, we noticed statistically significant differences between the results of the proteomic analysis corresponding to the samples.

We have described that there are qualitative, quantitative, and functional variations among the proteins included in the exosomes found from MSCs and activated MSCs^*^ (44). Thus, the comparison between cells studied in basal and in activated states shows that whereas the amounts of very significant common GO terms and MSC GO terms are in concordance, the results produced a significant variability and number of pathways modified in MSCs^*^ (44), and it demonstrates the intense metabolic change that these activated cell exosomes have suffered and the consequences after activation with radiation. Among the cluster representatives in MSCs^*^, we underline the leukocyte cell–cell adhesion, cell localization, and negative control of responses to activation and cell death. Several of these proteins are important elements of cell–cell or cell–matrix adhesion and include annexin and integrins. Among them, the presence of ANXA1 is very significant because it is always present in the exosomes secreted from MSCs^*^ and constantly absent in MSCs.

We have verified these findings using quantitative mRNA-PCR to measure the mRNA of this molecule in MSCs and MSCs^*^ and demonstrated that mRNA is dramatically induced in MSCs after irradiation, which supports the massive presence of ANXA1 in the exosomes released by MSCs^*^ (44). Especially relevant is the presence of ANXA 1 in the exosomes from activated MSCs^*^ and the absence of this protein in the conditioned medium separated from the non-irradiated MSCs.

During more than 30 years of research, annexins have been established as key elements in the control immune responses. The prototype member of this family, ANXA1, has been broadly accepted as an anti-inflammatory intermediary influencing migration and cellular reactions of various specialized cell types of the innate immune system (129). Nevertheless, it is now accepted that ANXA1 has extensive effects beyond the immune system with consequences in preserving homeostatic secretion, fetal development, the aging process, and development of several diseases such as cancer (130, 131).

Inflammation is a strongly controlled process, initiated after tissue damage or infection. If uncontrolled or unresolved, the inflammation itself can drive additional tissue destruction and cause persistent inflammatory disorders and autoimmunity with following deficiency of organ function. It is now clear that the control of inflammation is a functional process that appears during an acute inflammatory incident (132). Following cell activation and release, ANXA1 inhibits the accumulation of neutrophils in the tissue injured by numerous mechanisms; furthermore, ANXA1 promotes neutrophil apoptosis and takes actions on macrophages to stimulate the phagocytosis and the elimination of dead neutrophils (132, 133) and leads to the rapid restoration of tissue homeostasis. Inflammation outcome is regulated by numerous endogenous factors, involving fatty-acid-derived specialized pre-resolving mediators and protein, such as ANXA1 (134).

There is mounting evidence that ANXA1, and its mimetic peptides (135), may have a major function in mitigating ischemia–reperfusion injury-associated complications (136). Moreover, chronic inflammation in tumors is frequent and promotes tumor growth, progression, and metastatic spreading, as well as treatment resistance (137). Physical aberrancies of tumor vasculature comprise their chaotic organization, an enhanced interstitial pressure, an amplified solid stress, hypoxia, and a progressive contraction of solid tumors that are the physical barriers in tumors (138) and are inspiring new anti-cancer strategies aimed at targeting and normalizing the physical anomalies of these solid tumors (139).

On the other hand, the overexpression of this molecule has been reported in many cancers, although its clinical meaning is still controversial (140–142), which could be, in part, due to the localization of ANXA1 in the nuclear and cytoplasmic compartments, and also associated to the membrane (131). In fact, the expression level of ANXA1 is down-regulated in numerous types of cancer and is linked with metastasis, relapse, and poor prognosis (141, 143); ANXA1 is an endogenous inhibitor of NF-κB that may be stimulated in human cancer cells and in experimental mice models by powerful anti-inflammatory glucocorticoids and altered by non-steroidal anti-inflammatory drugs (143). In this context, ANXA1 has long been categorized as an anti-inflammatory molecule due to its influence over leukocyte-mediated immune responses (144).

Upon tissue damage, epithelial wound closure is a finely adjusted process detected in chronic inflammatory diseases related with non-healing wounds. In this process, ANXA1 is involved as a pre-resolving mediator (145). ANXA1 is a glucocorticoid-induced protein that is well-known to reproduce numerous anti-inflammatory effects of glucocorticoids and is implicated in the modulation of T-cell function and the adaptive immune response related to rheumatoid arthritis (146) and increasing data suggest that ANXA1, which act together with the formyl peptide receptor family, might have a major role in alleviating ischemia–reperfusion injury (136). ANXA1 interacts with p53 to co-regulate Bid expression and stimulate cell death after OGD/R via the caspase-3 pathway (147) and it has been described that ANXA1 is one of the molecules that is involved in p53-mediated radio-response and the abnormal expression of ANXA1 in nasopharyngeal carcinoma NPC might affect the apoptosis of tumor cells caused by ionizing radiation decreasing radiotherapeutic efficacy (148).

Recently, the function of ANXA1 in the therapy of acute radiation-induced lung injury has been analyzed and the mechanism of its action is investigated (149). The role of damage-associated molecular patterns in neuro-inflammation has been implicated in adverse neurological outcomes following lethal hemorrhagic shock and polytrauma. Data obtained in (150) provide new suggestion that appealing pro-resolving pharmacological approaches such as Annexin-A1 biomimetic peptides can effectively reduce neuro-inflammation and new data show a new multifaceted role for ANXA1 as a therapeutic and a prophylactic drug due to its capacity to stimulate endogenous pro-resolving, anti-thrombo-inflammatory circuits in cerebral ischemia–reperfusion injury (151). Finally, the chance of exploiting ANXA1 as a novel therapeutic molecule in diabetes and for treatment of microvascular disease has been announced (152).

## Conclusions

Considering all the information summarized in this review, we are convinced that, due to (i) the physical hallmarks and biological capabilities (153) that characterize neoplastic tissues, (ii) the physical–chemical tropism of MSCs (154), and (iii) the widespread functions of macromolecules, proteins, and exosomes, all these factors secreted by activated MSCs^*^ are able to reduce pro-tumorigenic and pro-metastatic signals released by tumors that influence the progression, growth, spread, and drug resistance of tumor cells.

However, additional study is required to find the cause of tumor cells forsaking malign phenotypes of cancer cells and returning to their normal state.

We have recently shown that clinical grade umbilical cord MSCs can be expanded, cryogenically stored, and reconstituted after batch release, maintaining their immunophenotype, and show good viability and activation by irradiation. Our study indicates that no toxic effects are produced by MSCs or pre-irradiated MSC^*^ inoculation. In addition, umbilical cord MSCs^*^ have never been detected in any studied organ at 90 days, indicating that these cells will not be present for a long time in a treated patient (manuscript in preparation).

In an attempt to take our basic and regulatory research to clinical practice, we proceeded to apply for the registration of the patent P201500022 and title “*Activated stem cells and medical uses*,” with the priority date of December 2014. Its international extension via PCT has the number PCT/ES2015/070951 (WO/2016/102735) and was published in June 2016.

Therefore, we conclude that umbilical cord mesenchymal cells combined with radiotherapy are adequate for therapeutic use in a clinical trial in patients with cancer due to the fact that increasing the therapeutic effect of radiotherapy on the tumors and possible metastatic foci improves the radiotherapy outcome.

## Author Contributions

JR and FO conceived and wrote the manuscript. VF performed most of the *in vitro* and *in vivo* experiments. FO'V performed immunohistochemistry. JE, RM, and IT critically revised the manuscript for important clinical observations. All the authors read and approved the final manuscript.

### Conflict of Interest

The authors declare that the research was conducted in the absence of any commercial or financial relationships that could be construed as a potential conflict of interest.

## References

[1] Singletary SE Allred C Ashley P Bassett LW Berry D Bland KI. Staging system for breast cancer: revisions for the 6th edition of the AJCC cancer staging manual. Surg Clin North Am. (2003) 83:803–19. 10.1016/S0039-6109(03)00034-3 12875597

[2] West CM McKay MJ Holscher T Baumann M Stratford IJ Bristow RG. Molecular markers predicting radiotherapy response: report and recommendations from an International Atomic Energy Agency technical meeting. Int J Radiat Oncol Biol Phys. (2005) 62:1264–73. 10.1016/j.ijrobp.2005.05.001 16029781

[3] Robnett TJ Machtay M Vines EF McKenna MG Algazy KM McKenna WG. Factors predicting severe radiation pneumonitis in patients receiving definitive chemoradiation for lung cancer. Int J Radiat Oncol Biol Phys. (2000) 48:89–94. 10.1016/S0360-3016(00)00648-9 10924976

[4] West CM Elliott RM Burnet NG. The genomics revolution and radiotherapy. Clin Oncol. (2007) 19:470–80. 10.1016/j.clon.2007.02.016 17419040

[5] Burnet NG Johansen J Turesson I Nyman J Peacock JH. Describing patients' normal tissue reactions: concerning the possibility of individualising radiotherapy dose prescriptions based on potential predictive assays of normal tissue radiosensitivity. Steering Committee of the BioMed2 European Union Concerted Action Programme on the development of predictive tests of normal tissue response to radiation therapy. Int J Cancer. (1998) 79:606–13. 10.1002/(SICI)1097-0215(19981218)79:6<606::AID-IJC9>3.0.CO;2-Y 9842969

[6] Lopez E Guerrero R Nunez MI del Moral R Villalobos M Martinez-Galan J. Early and late skin reactions to radiotherapy for breast cancer and their correlation with radiation-induced DNA damage in lymphocytes. Breast Cancer Res. (2005) 7:R690–8. 10.1186/bcr1277 16168114 PMC1242135

[7] Peacock JH de Almodovar MR McMillan TJ Steel GG. The nature of the initial slope of radiation cell survival curves. BJR Suppl. (1992) 24:57–60. 1290713

[8] Brown JM Carlson DJ Brenner DJ. The tumor radiobiology of SRS and SBRT: are more than the 5 Rs involved? Int J Radiat Oncol Biol Phys. (2014) 88:254–62. 10.1016/j.ijrobp.2013.07.022 24411596 PMC3893711

[9] Steel GG McMillan TJ Peacock JH. The 5Rs of radiobiology. Int J Radiat Biol. (1989) 56:1045–8. 10.1080/09553008914552491 2574214

[10] Fowler JF. The linear-quadratic formula and progress in fractionated radiotherapy. Br J Radiol. (1989) 62:679–94. 10.1259/0007-1285-62-740-679 2670032

[11] Fowler JF. 21 years of biologically effective dose. Br J Radiol. (2010) 83:554–68. 10.1259/bjr/31372149 20603408 PMC3473681

[12] Herskind C Ma L Liu Q Zhang B Schneider F Veldwijk MR. Biology of high single doses of IORT: RBE, 5 R's, and other biological aspects. Radiat Oncol. (2017) 12:24. 10.1186/s13014-016-0750-3 28107823 PMC5251326

[13] Marples B Joiner MC. The response of Chinese hamster V79 cells to low radiation doses: evidence of enhanced sensitivity of the whole cell population. Radiat Res. (1993) 133:41–51. 10.2307/3578255 8434112

[14] Mothersill C Seymour CB. Radiation-induced bystander effects–implications for cancer. Nat Rev Cancer. (2004) 4:158–64. 10.1038/nrc1277 14964312

[15] Mothersill CE Moriarty MJ Seymour CB. Radiotherapy and the potential exploitation of bystander effects. Int J Radiat Oncol Biol Phys. (2004) 58:575–9. 10.1016/j.ijrobp.2003.09.038 14751530

[16] Prise KM Schettino G Folkard M Held KD. New insights on cell death from radiation exposure. Lancet Oncol. (2005) 6:520–8. 10.1016/S1470-2045(05)70246-1 15992701

[17] Marples B Wouters BG Joiner MC. An association between the radiation-induced arrest of G2-phase cells and low-dose hyper-radiosensitivity: a plausible underlying mechanism? Radiat Res. (2003) 160:38–45. 10.1667/RR3013 12816521

[18] Gomez-Millan J Katz IS Farias Vde A Linares-Fernandez JL Lopez-Penalver J Ortiz-Ferron G. The importance of bystander effects in radiation therapy in melanoma skin-cancer cells and umbilical-cord stromal stem cells. Radiother Oncol. (2012) 102:450–8. 10.1016/j.radonc.2011.11.002 22169765

[19] Formenti SC Demaria S. Systemic effects of local radiotherapy. Lancet Oncol. (2009) 10:718–26. 10.1016/S1470-2045(09)70082-8 19573801 PMC2782943

[20] Formenti SC Demaria S. Radiation therapy to convert the tumor into an *in situ* vaccine. Int J Radiat Oncol Biol Phys. (2012) 84:879–80. 10.1016/j.ijrobp.2012.06.020 23078897 PMC3811126

[21] Dickey JS Redon CE Nakamura AJ Baird BJ Sedelnikova OA Bonner WM. H2AX: functional roles and potential applications. Chromosoma. (2009) 118:683–92. 10.1007/s00412-009-0234-4 19707781 PMC3094848

[22] Azzam EI de Toledo SM Little JB. Expression of CONNEXIN43 is highly sensitive to ionizing radiation and other environmental stresses. Cancer Res. (2003) 63:7128–35. 14612506

[23] Demaria S Coleman CN Formenti SC. Radiotherapy: changing the Game in Immunotherapy. Trends Cancer. (2016) 2:286–94. 10.1016/j.trecan.2016.05.002 27774519 PMC5070800

[24] Demaria S Golden EB Formenti SC. Role of local radiation therapy in cancer immunotherapy. JAMA Oncol. (2015) 1:1325–32. 10.1001/jamaoncol.2015.2756 26270858

[25] Azzam EI de Toledo SM Little JB. Oxidative metabolism, gap junctions and the ionizing radiation-induced bystander effect. Oncogene. (2003) 22:7050–7. 10.1038/sj.onc.1206961 14557810

[26] Goodhead DT. New radiobiological, radiation risk and radiation protection paradigms. Mutat Res. (2010) 687:13–6. 10.1016/j.mrfmmm.2010.01.006 20093132

[27] López E Núñez MI Guerrero MR del Moral R de Dios Luna J del Mar Rodríguez M. Breast cancer acute radiotherapy morbidity evaluated by different scoring systems. Breast Cancer Res Treat. (2002) 73:127–34. 10.1023/A:1015296607061 12088115

[28] Little JB. Genomic instability and bystander effects: a historical perspective. Oncogene. (2003) 22:6978–87. 10.1038/sj.onc.1206988 14557801

[29] Barcellos-Hoff MH Brooks AL. Extracellular signaling through the microenvironment: a hypothesis relating carcinogenesis, bystander effects, and genomic instability. Radiat Res. (2001) 156(5 Pt 2):618–27. 10.1667/0033-7587(2001)156[0618:ESTTMA]2.0.CO;2 11604083

[30] Burdak-Rothkamm S Rothkamm K Prise KM. ATM acts downstream of ATR in the DNA damage response signaling of bystander cells. Cancer Res. (2008) 68:7059–65. 10.1158/0008-5472.CAN-08-0545 18757420 PMC2528059

[31] Ivanov VN Zhou H Ghandhi SA Karasic TB Yaghoubian B Amundson SA. Radiation-induced bystander signaling pathways in human fibroblasts: a role for interleukin-33 in the signal transmission. Cell Signal. (2010) 22:1076–87. 10.1016/j.cellsig.2010.02.010 20206688 PMC2860693

[32] Luce A Courtin A Levalois C Altmeyer-Morel S Romeo PH Chevillard S. Death receptor pathways mediate targeted and non-targeted effects of ionizing radiations in breast cancer cells. Carcinogenesis. (2009) 30:432–9. 10.1093/carcin/bgp008 19126655 PMC2650794

[33] Seymour CB Mothersill C. Delayed expression of lethal mutations and genomic instability in the progeny of human epithelial cells that survived in a bystander-killing environment. Radiat Oncol Investig. (1997) 5:106–10. 10.1002/(SICI)1520-6823(1997)5:3<106::AID-ROI4>3.0.CO;2-1 9303065

[34] Lambin P van Stiphout RG Starmans MH Rios-Velazquez E Nalbantov G Aerts HJ. Predicting outcomes in radiation oncology–multifactorial decision support systems. Nat Rev Clin Oncol. (2013) 10:27–40. 10.1038/nrclinonc.2012.196 23165123 PMC4555846

[35] Lara PC Lopez-Penalver JJ Farias Vde A Ruiz-Ruiz MC Oliver FJ Ruiz de Almodovar JM. Direct and bystander radiation effects: a biophysical model and clinical perspectives. Cancer Lett. (2015) 356:5–16. 10.1016/j.canlet.2013.09.006 24045041

[36] Boustani J Grapin M Laurent PA Apetoh L Mirjolet C. The 6th R of radiobiology: reactivation of anti-tumor immune response. Cancers. (2019) 11:E860. 10.3390/cancers11060860 31226866 PMC6627091

[37] Ebert MA Suchowerska N Jackson MA McKenzie DR. A mathematical framework for separating the direct and bystander components of cellular radiation response. Acta Oncol. (2010) 49:1334–43. 10.3109/0284186X.2010.487874 20507257

[38] de Araujo Farias V O'Valle F Lerma BA Ruiz de Almodovar C Lopez-Penalver JJ Nieto A. Human mesenchymal stem cells enhance the systemic effects of radiotherapy. Oncotarget. (2015) 6:31164–80. 10.18632/oncotarget.5216 26378036 PMC4741595

[39] Curtis SB. Lethal and potentially lethal lesions induced by radiation–a unified repair model. Radiat Res. (1986) 106:252–70. 10.2307/3576798 3704115

[40] Lee RH Yoon N Reneau JC Prockop DJ. Preactivation of human MSCs with TNF-alpha enhances tumor-suppressive activity. Cell Stem Cell. (2012) 11:825–35. 10.1016/j.stem.2012.10.001 23142520

[41] Herskind C Wenz F Giordano FA. Immunotherapy combined with large fractions of radiotherapy: stereotactic radiosurgery for brain metastases-implications for intraoperative radiotherapy after resection. Front Oncol. (2017) 7:147. 10.3389/fonc.2017.00147 28791250 PMC5522878

[42] Golden EB Apetoh L. Radiotherapy and immunogenic cell death. Semin Radiat Oncol. (2015) 25:11–7. 10.1016/j.semradonc.2014.07.005 25481261

[43] Van der Meeren A Monti P Vandamme M Squiban C Wysocki J Griffiths N. Abdominal radiation exposure elicits inflammatory responses and abscopal effects in the lungs of mice. Radiat Res. (2005) 163:144–52. 10.1667/RR3293 15658889

[44] de Araujo Farias V O'Valle F Serrano-Saenz S Anderson P Andres E Lopez-Penalver J. Exosomes derived from mesenchymal stem cells enhance radiotherapy-induced cell death in tumor and metastatic tumor foci. Mol Cancer. (2018) 17:122. 10.1186/s12943-018-0867-0 30111323 PMC6094906

[45] Kim SM Oh JH Park SA Ryu CH Lim JY Kim DS. Irradiation enhances the tumor tropism and therapeutic potential of tumor necrosis factor-related apoptosis-inducing ligand-secreting human umbilical cord blood-derived mesenchymal stem cells in glioma therapy. Stem Cells. (2010) 28:2217–28. 10.1002/stem.543 20945331

[46] Manda G Isvoranu G Comanescu MV Manea A Debelec Butuner B Korkmaz KS. The redox biology network in cancer pathophysiology and therapeutics. Redox Biol. (2015) 5:347–57. 10.1016/j.redox.2015.06.014 26122399 PMC4501561

[47] Szeifert GT Salmon I Rorive S Massager N Devriendt D Simon S. Does gamma knife surgery stimulate cellular immune response to metastatic brain tumors? A histopathological and immunohistochemical study. J Neurosurg. (2005) 102(Suppl):180–4. 10.3171/sup.2005.102.s_supplement.0180 15662806

[48] Postow MA Callahan MK Barker CA Yamada Y Yuan J Kitano S. Immunologic correlates of the abscopal effect in a patient with melanoma. N Engl J Med. (2012) 366:925–31. 10.1056/NEJMoa1112824 22397654 PMC3345206

[49] Francois S Usunier B Forgue-Lafitte ME L'Homme B Benderitter M Douay L. Mesenchymal stem cell administration attenuates colon cancer progression by modulating the immune component within the colorectal tumor microenvironment. Stem Cells Transl Med. (2019) 8:285–300. 10.1002/sctm.18-0117 30451398 PMC6392393

[50] Prise KM O'Sullivan JM. Radiation-induced bystander signalling in cancer therapy. Nat Rev Cancer. (2009) 9:351–60. 10.1038/nrc2603 19377507 PMC2855954

[51] Nesslinger NJ Sahota RA Stone B Johnson K Chima N King C. Standard treatments induce antigen-specific immune responses in prostate cancer. Clin Cancer Res. (2007) 13:1493–502. 10.1158/1078-0432.CCR-06-1772 17332294

[52] Garnett CT Palena C Chakraborty M Tsang KY Schlom J Hodge JW. Sublethal irradiation of human tumor cells modulates phenotype resulting in enhanced killing by cytotoxic T lymphocytes. Cancer Res. (2004) 64:7985–94. 10.1158/0008-5472.CAN-04-1525 15520206

[53] Chakraborty M Abrams SI Coleman CN Camphausen K Schlom J Hodge JW. External beam radiation of tumors alters phenotype of tumor cells to render them susceptible to vaccine-mediated T-cell killing. Cancer Res. (2004) 64:4328–37. 10.1158/0008-5472.CAN-04-0073 15205348

[54] Formenti SC Lee P Adams S Goldberg JD Li X Xie MW. Focal irradiation and systemic TGFβ blockade in metastatic breast cancer. Clin Cancer Res. (2018) 24:2493–504. 10.1158/1078-0432.CCR-17-3322 29476019 PMC5999326

[55] Ohba K Omagari K Nakamura T Ikuno N Saeki S Matsuo I. Abscopal regression of hepatocellular carcinoma after radiotherapy for bone metastasis. Gut. (1998) 43:575–7. 10.1136/gut.43.4.575 9824589 PMC1727260

[56] Camphausen K Moses MA Menard C Sproull M Beecken WD Folkman J. Radiation abscopal antitumor effect is mediated through p53. Cancer Res. (2003) 63:1990–3. 10.1016/S0360-3016(02)03449-1 12702593

[57] Konoeda K. Therapeutic efficacy of pre-operative radiotherapy on breast carcinoma: in special reference to its abscopal effect on metastatic lymph-nodes. Nihon Gan Chiryo Gakkai Shi. (1990) 25:1204–14. 2398302

[58] Nobler MP. The abscopal effect in malignant lymphoma and its relationship to lymphocyte circulation. Radiology. (1969) 93:410–2. 10.1148/93.2.410 5822721

[59] Petrovic N Perovic J Karanovic D Todorovic L Petrovic V. Abscopal effects of local fractionated X-irradiation of face and jaw region. Strahlentherapie. (1982) 158:40–2. 7058543

[60] RaventosA An abscopal effect of x-ray upon mouse spleen weight. Radiat Res. (1954) 1:381–7. 10.2307/3570292 13186096

[61] Rees GJ Ross CM. Abscopal regression following radiotherapy for adenocarcinoma. Br J Radiol. (1983) 56:63–6. 10.1259/0007-1285-56-661-63 6185172

[62] Hiniker SM Chen DS Reddy S Chang DT Jones JC Mollick JA. A systemic complete response of metastatic melanoma to local radiation and immunotherapy. Transl Oncol. (2012) 5:404–7. 10.1593/tlo.12280 23323154 PMC3542835

[63] Stamell EF Wolchok JD Gnjatic S Lee NY Brownell I. The abscopal effect associated with a systemic anti-melanoma immune response. Int J Radiat Oncol Biol Phys. (2013) 85:293–5. 10.1016/j.ijrobp.2012.03.017 22560555 PMC3415596

[64] Ishiyama H Teh BS Ren H Chiang S Tann A Blanco AI. Spontaneous regression of thoracic metastases while progression of brain metastases after stereotactic radiosurgery and stereotactic body radiotherapy for metastatic renal cell carcinoma: abscopal effect prevented by the blood-brain barrier? Clin Genitourin Cancer. (2012) 10:196–8. 10.1016/j.clgc.2012.01.004 22409865

[65] Cotter SE McBride SM Yock TI. Proton radiotherapy for solid tumors of childhood. Technol Cancer Res Treat. (2012) 11:267–78. 10.7785/tcrt.2012.500295 22417062 PMC4527470

[66] Demaria S Pilones KA Formenti SC Dustin ML. Exploiting the stress response to radiation to sensitize poorly immunogenic tumors to anti-CTLA-4 treatment. Oncoimmunology. (2013) 2:e23127. 10.4161/onci.23127 23802063 PMC3661148

[67] Herter-Sprie GS Koyama S Korideck H Hai J Deng J Li YY. Synergy of radiotherapy and PD-1 blockade in Kras-mutant lung cancer. JCI Insight. (2016) 1:e87415. 10.1172/jci.insight.87415 27699275 PMC5033933

[68] Dovedi SJ Cheadle EJ Popple AL Poon E Morrow M Stewart R. Fractionated radiation therapy stimulates antitumor immunity mediated by both resident and infiltrating polyclonal T-cell populations when combined with PD-1 blockade. Clin Cancer Res. (2017) 23:5514–26. 10.1158/1078-0432.CCR-16-1673 28533222

[69] Pitroda SP Chmura SJ Weichselbaum RR. Integration of radiotherapy and immunotherapy for treatment of oligometastases. Lancet Oncol. (2019) 20:e434–42. 10.1016/S1470-2045(19)30157-3 31364595

[70] Pitroda SP Weichselbaum RR. Integrated molecular and clinical staging defines the spectrum of metastatic cancer. Nat Rev Clin Oncol. (2019) 16:581–8. 10.1038/s41571-019-0220-6 31092903

[71] Golden EB Chhabra A Chachoua A Adams S Donach M Fenton-Kerimian M. Local radiotherapy and granulocyte-macrophage colony-stimulating factor to generate abscopal responses in patients with metastatic solid tumours: a proof-of-principle trial. Lancet Oncol. (2015) 16:795–803. 10.1016/S1470-2045(15)00054-6 26095785

[72] Formenti SC Rudqvist NP Golden E Cooper B Wennerberg E Lhuillier C. Radiotherapy induces responses of lung cancer to CTLA-4 blockade. Nat Med. (2018) 24:1845–51. 10.1038/s41591-018-0232-2 30397353 PMC6286242

[73] Herrera FG Bourhis J Coukos G. Radiotherapy combination opportunities leveraging immunity for the next oncology practice. CA Cancer J Clin. (2017) 67:65–85. 10.3322/caac.21358 27570942

[74] Lai RC Yeo RW Lim SK. Mesenchymal stem cell exosomes. Semin Cell Dev Biol. (2015) 40:82–8. 10.1016/j.semcdb.2015.03.001 25765629

[75] Oliver L Hue E Sery Q Lafargue A Pecqueur C Paris F. Differentiation-related response to DNA breaks in human mesenchymal stem cells. Stem Cells. (2013) 31:800–7. 10.1002/stem.1336 23341263

[76] Matsuda S Nakagawa Y Kitagishi Y Nakanishi A Murai T. Reactive oxygen species, superoxide dimutases, and PTEN-p53-AKT-MDM2 signaling loop network in mesenchymal stem/stromal cells regulation. Cells. (2018) 7:36. 10.3390/cells7050036 29723979 PMC5981260

[77] Perez-Estenaga I Prosper F Pelacho B. Allogeneic mesenchymal stem cells and biomaterials: the perfect match for cardiac repair? Int J Mol Sci. (2018) 19:3236. 10.3390/ijms19103236 30347686 PMC6213975

[78] Decrock E Hoorelbeke D Ramadan R Delvaeye T De Bock M Wang N. Calcium, oxidative stress and connexin channels, a harmonious orchestra directing the response to radiotherapy treatment? Biochim Biophys Acta Mol Cell Res. (2017) 1864:1099–120. 10.1016/j.bbamcr.2017.02.007 28193563

[79] Chang PY Qu YQ Wang J Dong LH. The potential of mesenchymal stem cells in the management of radiation enteropathy. Cell Death Dis. (2015) 6:e1840. 10.1038/cddis.2015.189 26247725 PMC4558492

[80] Maziarz RT Devos T Bachier CR Goldstein SC Leis JF Devine SM. Single and multiple dose MultiStem (multipotent adult progenitor cell) therapy prophylaxis of acute graft-versus-host disease in myeloablative allogeneic hematopoietic cell transplantation: a phase 1 trial. Biol Blood Marrow Transplant. (2015) 21:720–8. 10.1016/j.bbmt.2014.12.025 25555450

[81] Klein D Schmetter A Imsak R Wirsdorfer F Unger K Jastrow H. Therapy with multipotent mesenchymal stromal cells protects lungs from radiation-induced injury and reduces the risk of lung metastasis. Antioxid Redox Signal. (2016) 24:53–69. 10.1089/ars.2014.6183 26066676

[82] Nicolay NH Lopez Perez R Saffrich R Huber PE. Radio-resistant mesenchymal stem cells: mechanisms of resistance and potential implications for the clinic. Oncotarget. (2015) 6:19366–80. 10.18632/oncotarget.4358 26203772 PMC4637291

[83] Lysaght T Campbell AV. Regulating autologous adult stem cells: the FDA steps up. Cell Stem Cell. (2011) 9:393–6. 10.1016/j.stem.2011.09.013 22056136

[84] Bernardo ME Cometa AM Locatelli F. Mesenchymal stromal cells: a novel and effective strategy for facilitating engraftment and accelerating hematopoietic recovery after transplantation? Bone Marrow Transplant. (2012) 47:323–9. 10.1038/bmt.2011.102 21552300

[85] Loebinger MR Janes SM. Stem cells as vectors for antitumour therapy. Thorax. (2010) 65:362–9. 10.1136/thx.2009.128025 20388765 PMC3401681

[86] Loebinger MR Sage EK Davies D Janes SM. TRAIL-expressing mesenchymal stem cells kill the putative cancer stem cell population. Br J Cancer. (2010) 103:1692–7. 10.1038/sj.bjc.6605952 21063402 PMC2994223

[87] Nicolay NH Liang Y Lopez Perez R Bostel T Trinh T Sisombath S. Mesenchymal stem cells are resistant to carbon ion radiotherapy. Oncotarget. (2015) 6:2076–87. 10.18632/oncotarget.2857 25504442 PMC4385837

[88] Skripcak T Belka C Bosch W Brink C Brunner T Budach V. Creating a data exchange strategy for radiotherapy research: towards federated databases and anonymised public datasets. Radiother Oncol. (2014) 113:303–9. 10.1016/j.radonc.2014.10.001 25458128 PMC4648243

[89] Murphy MB Moncivais K Caplan AI. Mesenchymal stem cells: environmentally responsive therapeutics for regenerative medicine. Exp Mol Med. (2013) 45:e54. 10.1038/emm.2013.94 24232253 PMC3849579

[90] Cotter SE Dunn GP Collins KM Sahni D Zukotynski KA Hansen JL. Abscopal effect in a patient with metastatic Merkel cell carcinoma following radiation therapy: potential role of induced antitumor immunity. Arch Dermatol. (2011) 147:870–2. 10.1001/archdermatol.2011.176 21768497

[91] Bergfeld SA Blavier L Declerck YA. Bone marrow-derived mesenchymal stromal cells promote survival and drug resistance in tumor cells. Mol Cancer Ther. (2014) 13:962–75. 10.1158/1535-7163.MCT-13-0400 24502925 PMC4000239

[92] Yagi H Kitagawa Y. The role of mesenchymal stem cells in cancer development. Front Genet. (2013) 4:261. 10.3389/fgene.2013.00261 24348516 PMC3842093

[93] Green DR. Cell competition: pirates on the tangled bank. Cell Stem Cell. (2010) 6:287–8. 10.1016/j.stem.2010.03.006 20362527

[94] de Araújo Farias V Linares-Fernandez JL Penalver JL Paya Colmenero JA Ferron GO Duran EL Human umbilical cord stromal stem cell express CD10 and exert contractile properties. Placenta. (2011) 32:86–95. 10.1016/j.placenta.2010.11.003 21126763

[95] López Peñalver JJ de Araujo Farías V López-Ramón MV Tassi M Oliver FJ Moreno-Castilla C Activated carbon cloth as support for mesenchymal stem cell growth and differentiation to osteocyte. Carbon. (2009) 47:3574–7. 10.1016/j.carbon.2009.08.016

[96] de Araújo Farias V Linares Fernández JL Sirés-Campos J López-Ramón MV Moreno-Castilla C Oliver FJ Growth and spontaneous differentiation of umbilical-cord stromal stem cells on activated carbon cloth. J Mater Chem B. (2013) 1:3359–68. 10.1039/c3tb20305k 32260926

[97] Rhee KJ Lee JI Eom YW Mesenchymal stem cell-mediated effects of tumor support or suppression. Int J Mol Sci. (2015) 16:30015–33. 10.3390/ijms161226215 26694366 PMC4691158

[98] Barcellos-de-Souza P Comito G Pons-Segura C Taddei ML Gori V Becherucci V. Mesenchymal stem cells are recruited and activated into carcinoma-associated fibroblasts by prostate cancer microenvironment-derived TGF-β1. Stem Cells. (2016) 34:2536–47. 10.1002/stem.2412 27300750

[99] Shi Y Du L Lin L Wang Y. Tumour-associated mesenchymal stem/stromal cells: emerging therapeutic targets. Nat Rev Drug Discov. (2017) 16:35–52. 10.1038/nrd.2016.193 27811929

[100] O'Malley G Heijltjes M Houston AM Rani S Ritter T Egan LJ. Mesenchymal stromal cells (MSCs) and colorectal cancer: a troublesome twosome for the anti-tumour immune response? Oncotarget. (2016) 7:60752–74. 10.18632/oncotarget.11354 27542276 PMC5312417

[101] Hass R von der Ohe J Ungefroren H. Potential role of MSC/cancer cell fusion and EMT for breast cancer stem cell formation. Cancers. (2019) 11:E1432. 10.3390/cancers11101432 31557960 PMC6826868

[102] Li W Ren G Huang Y Su J Han Y Li J. Mesenchymal stem cells: a double-edged sword in regulating immune responses. Cell Death Differ. (2012) 19:1505–13. 10.1038/cdd.2012.26 22421969 PMC3422473

[103] Ren G Zhao X Wang Y Zhang X Chen X Xu C. CCR2-dependent recruitment of macrophages by tumor-educated mesenchymal stromal cells promotes tumor development and is mimicked by TNFα. Cell Stem Cell. (2012) 11:812–24. 10.1016/j.stem.2012.08.013 23168163 PMC3518598

[104] Chowdhury R Webber JP Gurney M Mason MD Tabi Z Clayton A. Cancer exosomes trigger mesenchymal stem cell differentiation into pro-angiogenic and pro-invasive myofibroblasts. Oncotarget. (2015) 6:715–31. 10.18632/oncotarget.2711 25596732 PMC4359250

[105] Vieira de Castro J Gomes ED Granja S Anjo SI Baltazar F Manadas B. Impact of mesenchymal stem cells' secretome on glioblastoma pathophysiology. J Transl Med. (2017) 15:200. 10.1186/s12967-017-1303-8 28969635 PMC5625623

[106] Wu YL Li HY Zhao XP Jiao JY Tang DX Yan LJ. Mesenchymal stem cell-derived CCN2 promotes the proliferation, migration and invasion of human tongue squamous cell carcinoma cells. Cancer Sci. (2017) 108:897–909. 10.1111/cas.13202 28208216 PMC5448615

[107] Lobb RJ Lima LG Moller A. Exosomes: key mediators of metastasis and pre-metastatic niche formation. Semin Cell Dev Biol. (2017) 67:3–10. 10.1016/j.semcdb.2017.01.004 28077297

[108] Shin JW Son JY Raghavendran HR Chung WK Kim HG Park HJ. High-dose ionizing radiation-induced hematotoxicity and metastasis in mice model. Clin Exp Metast. (2011) 28:803–10. 10.1007/s10585-011-9411-y 21769700

[109] Hamalukic M Huelsenbeck J Schad A Wirtz S Kaina B Fritz G. Rac1-regulated endothelial radiation response stimulates extravasation and metastasis that can be blocked by HMG-CoA reductase inhibitors. PLoS ONE. (2011) 6:e26413. 10.1371/journal.pone.0026413 22039482 PMC3198428

[110] Di Modica M Regondi V Sandri M Iorio MV Zanetti A Tagliabue E. Breast cancer-secreted miR-939 downregulates VE-cadherin and destroys the barrier function of endothelial monolayers. Cancer Lett. (2017) 384:94–100. 10.1016/j.canlet.2016.09.013 27693459

[111] Peinado H Aleckovic M Lavotshkin S Matei I Costa-Silva B Moreno-Bueno G. Melanoma exosomes educate bone marrow progenitor cells toward a pro-metastatic phenotype through MET. Nat Med. (2012) 18:883–91. 10.1038/nm.2753 22635005 PMC3645291

[112] Hoshino A Costa-Silva B Shen TL Rodrigues G Hashimoto A Tesic Mark M. Tumour exosome integrins determine organotropic metastasis. Nature. (2015) 527:329–35. 10.1038/nature15756 26524530 PMC4788391

[113] Weidle UH Birzele F Kollmorgen G Ruger R. Long non-coding RNAs and their role in metastasis. Cancer Genomics Proteomics. (2017) 14:143–60. 10.21873/cgp.20027 28446530 PMC5420816

[114] Weidle UH Birzele F Kollmorgen G Ruger R. The multiple roles of exosomes in metastasis. Cancer Genomics Proteomics. (2017) 14:1–15. 10.21873/cgp.20015 28031234 PMC5267497

[115] Sun Z Shi K Yang S Liu J Zhou Q Wang G. Effect of exosomal miRNA on cancer biology and clinical applications. Mol Cancer. (2018) 17:147. 10.1186/s12943-018-0897-7 30309355 PMC6182840

[116] Le Large TYS Bijlsma MF Kazemier G van Laarhoven HWM Giovannetti E Jimenez CR. Key biological processes driving metastatic spread of pancreatic cancer as identified by multi-omics studies. Semin Cancer Biol. (2017) 44:153–69. 10.1016/j.semcancer.2017.03.008 28366542

[117] Dai J Escara-Wilke J Keller JM Jung Y Taichman RS Pienta KJ. Primary prostate cancer educates bone stroma through exosomal pyruvate kinase M2 to promote bone metastasis. J Exp Med. (2019) 216:2883. 10.1084/jem.20190158 31548301 PMC6888980

[118] Ostenfeld MS Jeppesen DK Laurberg JR Boysen AT Bramsen JB Primdal-Bengtson B. Cellular disposal of miR23b by RAB27-dependent exosome release is linked to acquisition of metastatic properties. Cancer Res. (2014) 74:5758–71. 10.1158/0008-5472.CAN-13-3512 25261234

[119] Baumgart S Holters S Ohlmann CH Bohle R Stockle M Ostenfeld MS. Exosomes of invasive urothelial carcinoma cells are characterized by a specific miRNA expression signature. Oncotarget. (2017) 8:58278–91. 10.18632/oncotarget.17619 28938555 PMC5601651

[120] Schwarzenbach H. Clinical relevance of circulating, cell-free and exosomal microRNAs in plasma and serum of breast cancer patients. Oncol Res Treat. (2017) 40:423–9. 10.1159/000478019 28683441

[121] Rager TM Olson JK Zhou Y Wang Y Besner GE. Exosomes secreted from bone marrow-derived mesenchymal stem cells protect the intestines from experimental necrotizing enterocolitis. J Pediatric Surg. (2016) 51:942–7. 10.1016/j.jpedsurg.2016.02.061 27015901 PMC4921266

[122] Chapel A Mohty M Trial Evaluating the Efficacy of Systemic Mesenchymal Stromal Cell (MSC) Injections for the Treatment of Severe and Chronic Radiotherapy-induced Abdomino-pelvic Complications (Pelvic Radiation Disease, PRD) Refractory to Standard Therapy (PRISME). ClinicalTrials.gov Identifier: NCT02814864.

[123] Moussa L Pattappa G Doix B Benselama SL Demarquay C Benderitter M. A biomaterial-assisted mesenchymal stromal cell therapy alleviates colonic radiation-induced damage. Biomaterials. (2017) 115:40–52. 10.1016/j.biomaterials.2016.11.017 27886554

[124] Bessout R Demarquay C Moussa L Rene A Doix B Benderitter M. TH17 predominant T-cell responses in radiation-induced bowel disease are modulated by treatment with adipose-derived mesenchymal stromal cells. J Pathol. (2015) 237:435–46. 10.1002/path.4590 26177977

[125] Van de Putte D Demarquay C Van Daele E Moussa L Vanhove C Benderitter M. Adipose-derived mesenchymal stromal cells improve the healing of colonic anastomoses following high dose of irradiation through anti-inflammatory and angiogenic processes. Cell Transplant. (2017) 26:1919–30. 10.1177/0963689717721515 29390877 PMC5802630

[126] Leibacher J Henschler R. Biodistribution, migration and homing of systemically applied mesenchymal stem/stromal cells. Stem Cell Res Ther. (2016) 7:7. 10.1186/s13287-015-0271-2 26753925 PMC4709937

[127] Bateman ME Strong AL Gimble JM Bunnell BA. Concise review: using fat to fight disease: a systematic review of nonhomologous adipose-derived stromal/stem cell therapies. Stem Cells. (2018) 36:1311–28. 10.1002/stem.2847 29761573

[128] Jung PY Ryu H Rhee KJ Hwang S Lee CG Gwon SY. Adipose tissue-derived mesenchymal stem cells cultured at high density express IFN-beta and TRAIL and suppress the growth of H460 human lung cancer cells. Cancer Lett. (2019) 440–441:202–10. 10.1016/j.canlet.2018.10.017 30393160

[129] Weyd H. More than just innate affairs - on the role of annexins in adaptive immunity. Biol Chem. (2016) 397:1017–29. 10.1515/hsz-2016-0191 27467753

[130] Guo C Liu S Sun MZ. Potential role of Anxa1 in cancer. Future Oncol. (2013) 9:1773–93. 10.2217/fon.13.114 24156336

[131] Boudhraa Z Bouchon B Viallard C D'Incan M Degoul F. Annexin A1 localization and its relevance to cancer. Clin Sci. (2016) 130:205–20. 10.1042/CS20150415 26769657

[132] Alessandri AL Sousa LP Lucas CD Rossi AG Pinho V Teixeira MM. Resolution of inflammation: mechanisms and opportunity for drug development. Pharmacol Ther. (2013) 139:189–212. 10.1016/j.pharmthera.2013.04.006 23583354

[133] Soehnlein O Lindbom L. Phagocyte partnership during the onset and resolution of inflammation. Nat Rev Inmunol. (2010) 10:427–39. 10.1038/nri2779 20498669

[134] Fredman G Tabas I. Boosting inflammation resolution in atherosclerosis: the next frontier for therapy. Am J Pathol. (2017) 187:1211–21. 10.1016/j.ajpath.2017.01.018 28527709 PMC5455064

[135] Fredman G Spite M. Specialized pro-resolving mediators in cardiovascular diseases. Mol Aspects Med. (2017) 58:65–71. 10.1016/j.mam.2017.02.003 28257820 PMC5716486

[136] Ansari J Kaur G Gavins FNE. Therapeutic potential of annexin A1 in ischemia reperfusion injury. Int J Mol Sci. (2018) 19:E1211. 10.3390/ijms19041211 29659553 PMC5979321

[137] Shalapour S Karin M. Immunity, inflammation, and cancer: an eternal fight between good and evil. J Clin Invest. (2015) 125:3347–55. 10.1172/JCI80007 26325032 PMC4588298

[138] Ivey JW Bonakdar M Kanitkar A Davalos RV Verbridge SS. Improving cancer therapies by targeting the physical and chemical hallmarks of the tumor microenvironment. Cancer Lett. (2016) 380:330–9. 10.1016/j.canlet.2015.12.019 26724680 PMC4919249

[139] Nicolas-Boluda A Silva AKA Fournel S Gazeau F. Physical oncology: new targets for nanomedicine. Biomaterials. (2018) 150:87–99. 10.1016/j.biomaterials.2017.10.014 29035739

[140] Han GH Lu KJ Huang JX Zhang LX Dai SB Dai CL. Association of serum annexin A1 with treatment response and prognosis in patients with esophageal squamous cell carcinoma. J Cancer Res Ther. (2018) 14(Supplement):S667–74. 10.4103/0973-1482.187297 30249885

[141] Raulf N Lucarelli P Thavaraj S Brown S Vicencio JM Sauter T. Annexin A1 regulates EGFR activity and alters EGFR-containing tumour-derived exosomes in head and neck cancers. Eur J Cancer. (2018) 102:52–68. 10.1016/j.ejca.2018.07.123 30142511

[142] Vidotto A Polachini GM de Paula-Silva M Oliani SM Henrique T Lopez RVM. Differentially expressed proteins in positive versus negative HNSCC lymph nodes. BMC Med Genomics. (2018) 11:73. 10.1186/s12920-018-0382-6 30157864 PMC6114741

[143] Zhang Z Huang L Zhao W Rigas B. Annexin 1 induced by anti-inflammatory drugs binds to NF-κB and inhibits its activation: anticancer effects *in vitro* and *in vivo*. Cancer Res. (2010) 70:2379–88. 10.1158/0008-5472.CAN-09-4204 20215502 PMC2953961

[144] Sheikh MH Solito E. Annexin A1: uncovering the many talents of an old protein. Int J Mol Sci. (2018) 19:E1045. 10.3390/ijms19041045 29614751 PMC5979524

[145] Leoni G Nusrat A. Annexin A1: shifting the balance towards resolution and repair. Biol Chem. (2016) 397:971–9. 10.1515/hsz-2016-0180 27232634 PMC5361892

[146] Kao W Gu R Jia Y Wei X Fan H Harris J. A formyl peptide receptor agonist suppresses inflammation and bone damage in arthritis. Br J Pharmacol. (2014) 171:4087–96. 10.1111/bph.12768 24824742 PMC4243981

[147] Li X Zhao Y Xia Q Zheng L Liu L Zhao B. Nuclear translocation of annexin 1 following oxygen-glucose deprivation-reperfusion induces apoptosis by regulating Bid expression via p53 binding. Cell Death Dis. (2016) 7:e2356. 10.1038/cddis.2016.259 27584794 PMC5059862

[148] Zeng GQ Cheng AL Tang J Li GQ Li MX Qu JQ. Annexin A1: a new biomarker for predicting nasopharyngeal carcinoma response to radiotherapy. Med Hypotheses. (2013) 81:68–70. 10.1016/j.mehy.2013.04.019 23660133

[149] Han G Lu K Xu W Zhang S Huang J Dai C. Annexin A1-mediated inhibition of inflammatory cytokines may facilitate the resolution of inflammation in acute radiation-induced lung injury. Oncol Lett. (2019) 18:321–9. 10.3892/ol.2019.10317 31289503 PMC6540105

[150] Ma Q Zhang Z Shim JK Venkatraman TN Lascola CD Quinones QJ. Annexin A1 bioactive peptide promotes resolution of neuroinflammation in a rat model of exsanguinating cardiac arrest treated by emergency preservation and resuscitation. Front Neurosci. (2019) 13:608. 10.3389/fnins.2019.00608 31258464 PMC6587399

[151] Senchenkova EY Ansari J Becker F Vital SA Al-Yafeai Z Sparkenbaugh EM. Novel role for the AnxA1-Fpr2/ALX signaling axis as a key regulator of platelet function to promote resolution of inflammation. Circulation. (2019) 140:319–35. 10.1161/CIRCULATIONAHA.118.039345 31154815 PMC6687438

[152] Purvis GSD Solito E Thiemermann C. Annexin-A1: therapeutic potential in microvascular disease. Front Immunol. (2019) 10:938. 10.3389/fimmu.2019.00938 31114582 PMC6502989

[153] Hanahan D Weinberg RA. Hallmarks of cancer: the next generation. Cell. (2011) 144:646–74. 10.1016/j.cell.2011.02.013 21376230

[154] Thomas JG Parker Kerrigan BC Hossain A Gumin J Shinojima N Nwajei F. Ionizing radiation augments glioma tropism of mesenchymal stem cells. J Neurosurg. (2018) 128:287–95. 10.3171/2016.9.JNS16278 28362237 PMC6008155

